# Morphological and Molecular Changes in the Cortex and Cerebellum of Immunocompetent Mice Infected with Zika Virus

**DOI:** 10.3390/v15081632

**Published:** 2023-07-27

**Authors:** Aura Caterine Rengifo, Jorge Rivera, Diego Alejandro Álvarez-Díaz, Julián Naizaque, Gerardo Santamaria, Sheryll Corchuelo, Claudia Yadira Gómez, Orlando Torres-Fernández

**Affiliations:** 1 Grupo de Morfología Celular, Dirección de Investigación en Salud Pública, Instituto Nacional de Salud (INS), Avenue 26 No. 51-20–Zone 6 CAN, Bogotá 111321, Colombia; jrivera@ins.gov.co (J.R.); daalvarezd@unal.edu.co (D.A.Á.-D.); jnaizaque@ins.gov.co (J.N.); biojulmilger@gmail.com (G.S.); sheryll.corchuelo@gmail.com (S.C.); cgomez@ins.gov.co (C.Y.G.); otorresf@ins.gov.co (O.T.-F.); 2 Genómica de Microorganismos Emergentes, Dirección de Investigación en Salud Pública, Instituto Nacional de Salud (INS), Avenue 26 No. 51-20–Zone 6 CAN, Bogotá 111321, Colombia

**Keywords:** neuropathogenesis, Zika virus, cerebral cortex, cerebellum, neurodevelopmental markers

## Abstract

Zika virus (ZIKV) disease continues to be a threat to public health, and it is estimated that millions of people have been infected and that there have been more cases of serious complications than those already reported. Despite many studies on the pathogenesis of ZIKV, several of the genes involved in the malformations associated with viral infection are still unknown. In this work, the morphological and molecular changes in the cortex and cerebellum of mice infected with ZIKV were evaluated. Neonatal BALB/c mice were inoculated with ZIKV intraperitoneally, and the respective controls were inoculated with a solution devoid of the virus. At day 10 postinoculation, the mice were euthanized to measure the expression of the markers involved in cortical and cerebellar neurodevelopment. The infected mice presented morphological changes accompanied by calcifications, as well as a decrease in most of the markers evaluated in the cortex and cerebellum. The modifications found could be predictive of astrocytosis, dendritic pathology, alterations in the regulation systems of neuronal excitation and inhibition, and premature maturation, conditions previously described in other models of ZIKV infection and microcephaly.

## 1. Introduction

Knowledge on the pathogenesis of Zika virus (ZIKV) is scarce, although its isolation was achieved almost 70 years ago from the blood of a macaque from the Zika forest in Uganda [1,2]. Before 2007, reports of infection by this virus had been sporadic, and it was estimated that 80% of infections were asymptomatic. The clinical picture of ZIKV infection resembles that generated by the dengue and chikungunya viruses, manifesting with a fever, headache, arthralgia, myalgia, and maculopapular rash, a set of symptoms that make differential diagnosis difficult [3,4]. This symptomatology did not generate a significant impact on public health until the outbreaks reported in French Polynesia and Brazil in 2014 and 2015, respectively, where the appearance of neurological signs associated with ZIKV infection, such as Guillain–Barré autoimmune syndrome and microcephaly, were identified, with the latter anomaly mainly reported in Brazil and the Americas [1,5].

Most of the neurological associations with ZIKV were of a spatio-temporal type and did not exclude the participation of other flaviviruses. However, the first case report confirmed the presence of the virus in the brain of a fetus with microcephaly from a mother infected by Zika [6] and in the amniotic fluid of two women with children who presented malformations [7]. These studies suggested transplacental transmission and the possible neurotropism of ZIKV in humans [8]. This hypothesis was later confirmed by different studies published in humans and in murine models that simulated vertical infection [9,10,11,12].

The appearance of neonatal microcephaly, along with other neurodevelopmental disorders associated with ZIKV infection, sparked global alarm in late 2015 [13]. However, microcephaly is only one condition within the broad spectrum of fetal development disorders related to the virus; this condition is known as congenital Zika syndrome because it can be accompanied by dystrophic calcifications in various parts of the fetal brain [14], cognitive impairment [15], and eye tissue damage with visual impairment [6,16,17], among other signs and symptoms.

In vitro studies on ZIKV neuropathogenesis using neural stem cells, radial glia, and organoids have indicated that the virus can alter the cell cycle through dysregulation or arrest, and increase apoptosis [18,19,20,21] and alter pathways that regulate autophagy [12]. Additionally, there is an imbalance in the genes involved in the proliferation and differentiation of neural progenitor cells, with changes that promote cell differentiation [22], and in the rate of infection by stimulating autophagy pathways [12]. In these biomodels and in some in vivo tests, the viral proteins in ZIKV, i.e., NS4A and NS5, have been shown to cause the dysregulation of proteins involved in neurogenesis and neuronal migration, for example, doublecortin (DCX) [23], a protein responsible for cortical stratification [24,25].

Most studies on altered gene expression during ZIKV infection have been carried out in vitro models [21,26,27,28,29]. These studies have provided great value for bettering the understanding on viral neuropathogenesis, but do not recapitulate all the interactions of the extracellular matrix and the physiological microenvironment [30]. As an alternative to overcome this disadvantage, animal models, such as mice, hamsters, and nonhuman primates, have been used [31].

Mice are the most commonly used model to study viral infections in humans [32], and neonatal mice are optimal for evaluating neurodevelopment because they present events equivalent and parallel to the prenatal neurodevelopment of humans comparable to week 23 of the gestation period [33,34,35,36]. In both events, the subventricular zone is expanded to promote cortical development, multiple layers of the cerebral cortex are consolidated, action potentials are produced in the retina, and the optic nerve experiences significant growth and axon appearance [33]. In addition to the above, 1–3 days postnatally in both mice and humans, the blood–brain barrier is still maturing despite being present [37].

Adult mouse models of ZIKV infection are usually performed after blocking or attenuating IFN α/β signaling [10,38,39], as is the case with mice lacking the interferon receptor lfnar1−/− and triple knockout (TKo) mice for type I interferon regulatory factors, such as Irf3−/− Irf5−/− Irf7−/− Irf7−/−. Studies with different routes of inoculation in these mice and one-week-old immunocompetent mice have shown that ZIKV inoculated by subcutaneous (SC), intraperitoneal (IP), and intravenous (IV) pathways induce neurological signs associated with the disease [10,40,41], as well as damage in areas related to learning and memory processes [42]. The need to use this type of model reflects the inability of ZIKV to inhibit IFN α/β signaling or effector function efficiently; a finding similar to that observed with dengue virus [10].

In humans, ZIKV blocks signaling to the type I interferon through degradation of the STAT-2 factor through the replication protein NS5, while in mice, such degradation is inefficient with increasing age [43,44], especially when the inoculation is peripheral and performed in a range above the first week of age, as observed with other flaviviruses, such as dengue [45], so the use of neonatal mice turns out to be an excellent model when working with immunocompetent mice [33,46].

Embryonic or prenatal mouse models with ZIKV have allowed testing on the vertical and sexual transmission of the virus and its association with microcephaly or congenital malformations [39]. In these models, other routes of inoculation have been carried out, such as intrauterine inoculation of the mother, and the intra-amniotic or intracerebral (IC) route of the fetuses. All of these inoculations must be performed via laparoscopic surgery; however, when inoculation is performed by the intraventricular pathway, the mice do not survive after 2 to 3 days post-birth [18,47].

The intravaginal route has also been examined in the prenatal ZIKV model. Here, when the inoculation is performed at an embryonic stage, before the formation of the neural tube, the phase known as neurulation [48,49], the malformations are more evident, and there is even a risk of abortion [11,39], so inoculations before this stage do not allow for the simulation of the spectrum of morphological and molecular changes present during ZIKV pathogenesis.

Immunocompromised adult or prenatal mice [10,11,39], or immunocompetent neonatal mice, have been used for ZIKV studies, the latter being a model that is easy to obtain and manage because it requires fewer interventions to simulate the effects that occur during flavivirus infection. In addition, these mice have a poor response to some types of interferon and great permeability of the blood–brain barrier, two conditions that can favor fatal outcomes or the presence of fetal malformations caused by ZIKV infection [18,33,50,51,52,53].

Peripheral inoculations of ZIKV, such as intradermal (ID), IP and IV pathways, have allowed the testing of viral neurotropism, i.e., the capacity of flavivirus invasion and replication in the nervous system [41,54,55,56,57]. These inoculation routes favor the simulation of postnatal models of infection closer to the natural transmission pathway of ZIKV related to the models of infection obtained by IC, a classic inoculation route used for isolation and replication of the flavivirus, but that generates massive encephalic damage, high lethality, and severe neuronal death [45,50].

Considering the great advantages of using neonatal mice as models of Zika infection, in this study, we evaluated the morphological changes and the dysregulation of the neurodevelopmental genes in the cortex and cerebellum of immunocompetent neonatal mice inoculated with ZIKV, using an IP pathway.

## 2. Materials and Methods

### 2.1. Ethical Considerations

The experimental procedures using animals in this study were approved by the ethics committee of the Instituto Nacional de Salud–INS, Bogotá-Colombia (Minute No. 4, 19 May 2016). The current technical and ethical recommendations in Colombia and the international guidelines on the care and use of laboratory animals were followed [58], as were the guidelines in the European directive [59]. The biosafety and pathogen management standards by the INS and the Biosafety in Microbiological and Biomedical Laboratories (BMBL) of the CDC-NIH were also followed [60].

### 2.2. Generation of the Viral Stock

Vero cell monolayers (ATCC^®^ CCL-81) maintained in minimal essential medium (MEM) (Gibco, Grand Island, NY, USA), supplemented with 1% HEPES (Gibco, Bleiswijk, The Netherlands), 1% sodium bicarbonate, and 2% fetal bovine serum (FBS) (Gibco, Grand Island, NY, USA), were infected with ZIKV (MH544701.1). This strain was obtained from a patient positive for ZIKV at nine weeks of pregnancy; the processes for obtaining and purifying the virus were previously reported by our working group [41].

After five days of inoculation, the cell supernatants were collected, centrifuged at 300× *g* for 5 min and filtered through a membrane with a pore size of 0.22 µm to remove cellular debris. The viral titer was determined using plaque lysis tests in Vero cells, with six dilutions at a factor of 10 and subsequent analysis on the fifth day postinoculation (dpi).

### 2.3. Obtaining Mice Infected with ZIKV for Histological Studies and Differential Expression Assays

BALB/c mice of both sexes were maintained in the vivarium at the Instituto Nacional de Salud, Bogotá-Colombia, which has colonies with this strain from the Charles River Laboratory (Charles River Laboratories, Wilmington, MA, USA). Male and female adult mice were mated separately in different cages to obtain neonatal mice. The mice were kept in racks with a floor area of 516 cm^2^ and an internal height of 20 cm (ZyfoneTM, Lab Products Inc., Seaford, DE, USA), with controlled conditions for the temperature (23 °C +/− 1 °C) and humidity (55% +/− 10%), and access to food and water ad libitum.

The control and infected experimental groups came from different litters and were kept in separate racks. A total of 8 litters, each between 5 and 7 mice, were used with their mothers. Ten 1-day postnatal BALB/c mice (2 litters) were inoculated intraperitoneally with 40 µL of ZIKV (7.17 × 105 PFU/mL), and the respective controls (10 mice) were inoculated with a virus-free solution.

The clinical signs were monitored daily. The infected mice and controls were euthanized in a CO_2_ chamber at 10 dpi to minimize suffering [61]. The brains were then removed and placed in a frozen gel pack for dissection of the cortex and cerebellum. One hemisphere of each brain was placed in a vial with RNALater^®^ (Sigma Aldrich, Merck, Darmstadt, Germany) for up to one week; then, the reagent was removed, and the samples were stored at −80 °C for subsequent differential expression assays. The other hemisphere was fixed in 4% paraformaldehyde (PFA) for histological assays (hematoxylin and eosin (H&E), von Kossa staining, and immunohistochemistry (IHC) techniques.

To evaluate the implications of ZIKV infection on neurodevelopment, the following markers, which are involved in this process, were assessed: centrosomal protein 152 (CEP-152), chloride voltage-gated channel 2 (CLCN2), and glial fibrillary acidic protein (GFAP), microtubule-associated protein 2 (MAP-2), neuronal nuclear protein (NeuN), calbindin (Calb), parvalbumin (Parv), doublecortin (DCX), the β form of the S100 protein (S100β), cadherin 20 (CDH-20), reelin, and nestin. Alterations in the protein immunoreactivity and mRNA of the infected mice and negative controls (mock) were evaluated using IHC and differential expression tests.

### 2.4. Detection of ZIKV by qRT-PCR and Quantification of the Viral Load

Total RNA was extracted from the cortex and cerebellum using the miRNeasy^®^ Mini Kit (Qiagen, Hilden, Germany), following the manufacturer’s instructions. For all samples, the presence of ZIKV RNA was evaluated using real-time PCR, following the methodology previously standardized by our working group [62], i.e., using the SuperScript^®^ III one-step RT-PCR kit (Invitrogen™ Life Technologies, Carlsbad, CA, USA) and the following reaction conditions: a reaction buffer containing 3 mM magnesium sulfate (MgSO_4_), 0.2 mM of each dNTP, 0.21 μM of each primer, and 0.15 μM RNA. The total RNA template volume was 2 µL, and the reaction was brought up to a final volume of 10 µL. All samples were evaluated in duplicate. For these assays, we used the thermocycler Applied Biosystems 7500 Fast Dx Real-time PCR with the following conditions: reverse transcription at 50 °C for 30 min, enzyme activation at 95 °C for 2 min, and 45 cycles of 95 °C for 15 s and 60 °C for 1 min for hybridization and extension.

The number of viral copies was determined with a standard curve with a dilution factor of 10, using a DNA construct designed by our working group as a reference sample [62]. This construct was synthesized by Macrogen (Seoul, Republic of Korea) and transcribed into RNA using the HiScribe™ T7 High Yield RNA Synthesis kit (New England Biolabs, MA, USA). The calculations were performed following the protocol provided by Applied Biosystems [63]. For this, the Ct values obtained in the viral detection PCR were interpolated using the standard curve of the RNA construct of a known concentration. The final value was expressed as viral copy number/nanogram of the total RNA.

### 2.5. Histological Analysis and Immunohistochemical Detection of ZIKV

For the histological analysis, the cortex and cerebellum tissues from the hemispheres stored in 4% PFA were treated in a histoprocessor and embedded in paraffin. Coronal sections of the cortex and sagittal sections of the cerebellum ranging from 4 to 7 µm were prepared for H&E staining and the detection of calcifications using the von Kossa method [64]. Finally, the sections were dehydrated with ethanol in ascending concentrations and rinsed with xylol, after which they were mounted on slides with Entellan™.

For the immunohistochemical assays of ZIKV and neurodevelopmental markers, ten 1-day postnatal BALB/c mice (2 litters) were inoculated with the same viral dose of ZIKV, previously described for the differential expression studies. For the respective negative controls (mock), ten 1-day postnatal BALB/c mice (2 litters) were inoculated with a virus-free solution. When the mice showed advanced signs of infection (10 dpi), they were euthanized following the recommendations for immunohistochemical studies of the central nervous system (CNS) [65,66], which consists of fixing the brains via intracardiac perfusion.

The mice were anesthetized with an IP injection of 0.05 mL of 30% chloral hydrate (Merck), and then initially perfused with phosphate buffer saline (PBS) at pH 7.3 for 3 min and then with a 4% PFA fixative solution. After completing the perfusion with the fixative, the mouse was perfused with PBS for 1 min, after which the brain was immediately extracted and stored in the same fixation solution at 4 °C. The brain was separated from the cerebellum from the most caudal region of the cerebral hemispheres, isolating the brainstem attached to the cerebellum. The study areas comprised the cerebral cortex: primary motor cortex (M1), secondary motor cortex (M2), and primary somatosensory cortex, according to the Paxinos [67] and Franklin [68] mouse atlases. The study of the cerebellum mainly included the evaluation of the folia, constituted by the cortex of the cerebellum [67].

Half of the perfused brains from the infected and mock mice were embedded in paraffin to obtain 4-µm sagittal sections of a complete hemisphere in a microtome (Leica, JUNG RM2045). For the other half of the brains, 50-µm coronal cortex and sagittal sections of the cerebellum were made in a vibratome (Leica, VT1000 S, Wetzlar, Germany). Both sets of sections (microtome and vibratome) were used for the immunohistochemical studies on the neurodevelopmental markers, previously evaluated by qRT-PCR. The immunostaining assays were performed for the markers that could be evaluated by this technique, using the antibody concentrations described in Table 1, with the methodologies previously standardized by our work group [56,69,70].

The immunostainings were performed simultaneously on the controls and infected mice, and the images were captured with the same light intensity. The staining level for each marker evaluated was quantified by optical densitometry analysis in grayscale microphotographs using the software Photoshop 2021 v22.3.1. This analysis estimated the average gray tone of the reaction and allowed the comparison of densitometries between tissues from the control and the ZIKV-infected animals.

In all the perfused brains, the presence of the viral antigen was always confirmed for the groups infected with ZIKV using an anti-ZIKV antibody kindly provided by the CDC’s Division of Vector-Borne Diseases [71] and using an antibody generated by our working group.

### 2.6. Differential Expression Assays

Using the tissues stored in RNALater^®^ (Sigma Aldrich, Merck, Darmstadt, Germany and described in Section 2.3), the total RNA was purified from the cortex and cerebellum with the RNeasy^®^ Lipid Tissue kit (Qiagen, Hilden, Germany), following the manufacturer’s instructions, including the on-column RQ1 DNase digestion. The first strand cDNA was synthesized using random hexamer primers (5 µM) and 1 µg of total RNA with the GoScript reverse transcription system (Promega, Madison, WI, USA), following the manufacturer’s recommendations. The real-time qPCR assays to evaluate the expression of the genes described above were performed with four biological replicates for each treatment (infected and mock groups) in triplicate reactions, using GAPDH as the reference gene. All the reactions were performed using the Luna^®^ Universal qPCR Master Mix system (New England Biolab, Ipswich, MI, USA) and the primers and probes described in Table 2. The primers were designed using the PrimerSelect software (Lasergene version 7.2.1) or the sequences reported by other authors were used.

The total volume per reaction was 10 µL: 1.4 µL of cDNA (~22.5 ng), 5 µL of Luna^®^ Universal qPCR Master Mix (New England Biolab), and 2 µL of 5 µM primers. The following thermocycler conditions were used: 95 °C for 1 min for 1 cycle; 95 °C for 15 s and 60 °C for 30 s for 40 cycles; 60 °C for 5 min for 1 cycle; and 95 °C for 15 s and 60 °C for 1 min for 1 cycle, after amplification for the melting curve analysis from 60 °C to 95 °C.

The PCR curves were analyzed using Applied Biosystems 7500 v2.0.6 software. The working RNA concentration ranges were established using standard curves at different cDNA synthesis dilutions, and the efficiency per run was calculated using LinRegPCR software (2014.x) [75]. To estimate the relative gene expression, the Livak comparative Ct method [76] with the ExpressionSuite v 1.1 software and the Gene Expression Ct’s Difference (GED) method, using the average and individual efficiency [77], were used.

### 2.7. Network Analysis

The Biological Network Gene Ontology tool (BiNGO) and the ClueGO+Cluepedia plugins in the Cytoscape software were used for construction of the interaction networks with the genes dysregulated in the cerebellum and cortex of ZIKV-infected BALB/c mice. BiNGO was used to visualize the gene regulatory network through GO term enrichment analysis, including the biological process, cellular component, and molecular function. The disease dysregulated genes regulatory network was constructed using ClueGO+Cluepedia with ENCODE data for human diseases [78].

### 2.8. Statistical Analysis

For the statistical analyses, hypothesis contrast tests were performed using the parametric Student’s *t* test for those data with a normal distribution based on bias and kurtosis, normality tests, goodness-of-fit tests, and homoscedasticity tests. Data that did not meet these criteria were analyzed using a nonparametric test, i.e., Wilcoxon–Mann–Whitney U test in Statgraphics Centurion XVI version 16.1.03.

## 3. Results

### 3.1. Observation of Signs of Illness

The first signs of disease associated with ZIKV infection were observed at day 6 dpi; the infected mice showed stagnation in weight with respect to the controls (Figure S1). At 7 dpi, the infected mice exhibited group isolation, piloerection, tremor, uncoordinated gait possibly associated with ataxia, low activity, hypersensitivity to touch, and hypotonia. These signs were progressively accentuated until day 10 dpi, when the mice presented prostration or paralysis in the two hind limbs; euthanasia was carried out to minimize suffering and promote animal welfare.

### 3.2. Histological Findings

#### 3.2.1. Identification of Calcifications

**Figure 1:**
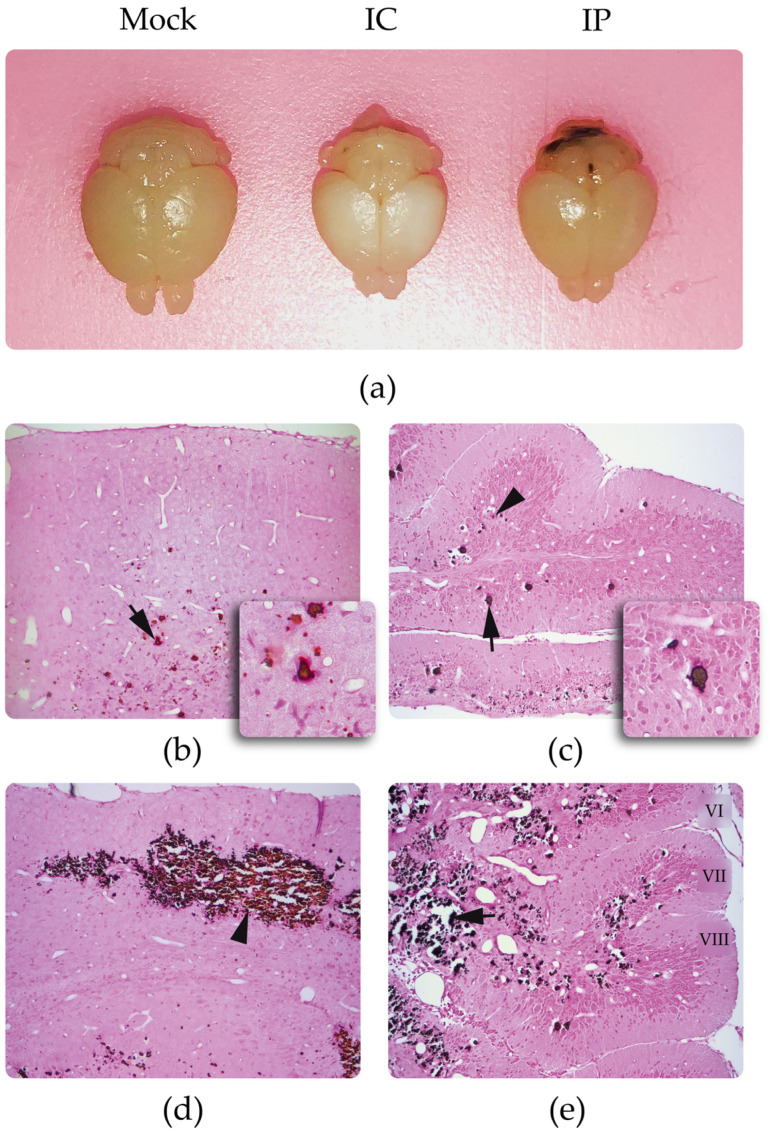
Von Kossa silver staining and eosin staining in Zika-infected mice: (**a**) Macroscopic image of 10 dpi control (mock) and infected brains inoculated intracerebrally (IC) and intraperitoneally (IP) (note the whitish appearance, mainly in the brain of the animals IC inoculated). The brain infected by the IC route is only shown to provide a clearer illustration of the macroscopic appearance of calcifications, but this route of inoculation was not the subject of this study; (**b**) anterior frontal cerebral cortex with positive staining for calcium (brown color) present in the cell soma of the infragranular layers (arrow); (**c**) cerebellum with positive staining for calcium in the Purkinje neurons (arrow) and granular cells (arrowhead); (**d**) panoramic image of the posterior frontal cortex at the level of the hippocampus, with a higher density of cells positive for calcium between layers III and V, note that calcifications are mainly seen in the infragranular layers of the cortex; (**e**) calcium-positive cells in the inner granular layer, i.e., folia VI, VII and VIII of the cerebellum. Note tissue damage in the calcified area. 20X.

In a macroscopic view of the brains of the infected mice, there were small areas in the cortex and cerebellum with certain whitish tones (Figure 1a). When preparing the histological sections of the brain, a brittle appearance with a loss of consistency was observed, indicating possible calcifications. The presence of calcium deposits in the cortex and in the cerebellum was confirmed by von Kossa silver staining, with a greater presence in frontal cortex and folia VI, VII, and VIII of cerebellum (Figure 1b–e).

#### 3.2.2. H&E Staining

**Figure 2:**
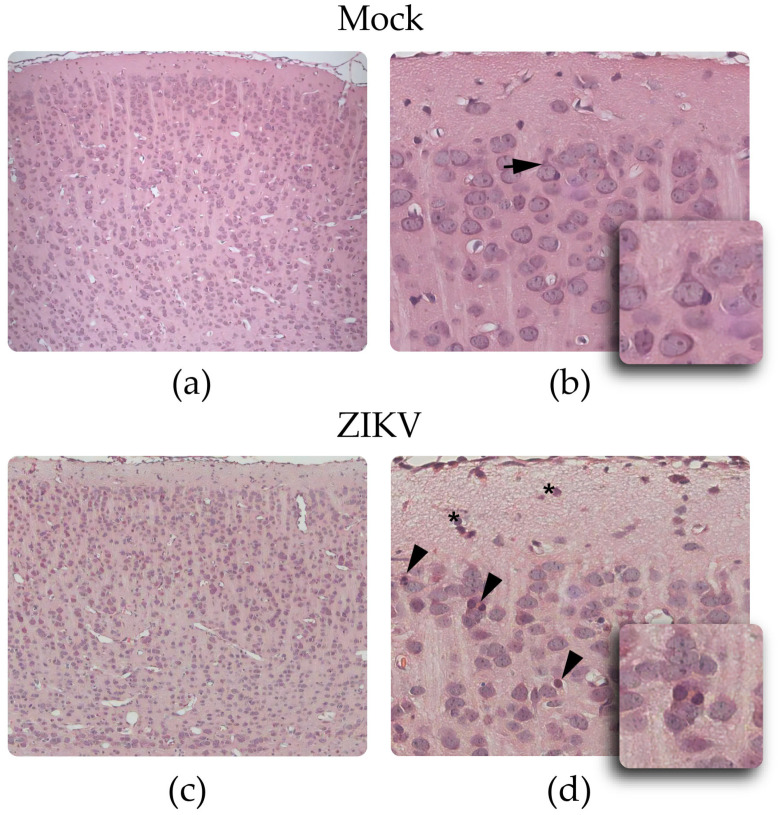
Histological findings in the cerebral cortex of control and Zika-infected mice: (**a**,**b**) control mouse and (**c**,**d**) infected mouse. (**b**) Detail from a control mouse, highlighting a greater number of large neurons and an apical dendrite directed toward the pia mater (arrow). (**d**) Detail from an infected mouse, where fewer neurons, a greater number of glial cells in layer I (asterisks) and pyknotic bodies (arrowhead) are observed. Images (**a**,**c**) at 20X, (**b**,**d**) at 80X.

The H&E staining revealed a uniform cellular distribution pattern in all the layers of the cerebral cortex from the controls (Figure 2a,b), and a more dispersed and less aligned cellular organization in the infected mice (Figure 2c,d). Large neurons with prominent and slightly stained nuclei were observed in the cortices from the mock group, with diameters between 50 and 108 µm, with cortical layers relatively aligned and close to each other, and with apical dendrites directed toward the pia mater. Other cells with small intensely basophilic nuclei, with no apparent cytoplasm around them, corresponding to glial cells, were also observed (Figure 2d).

In the group of infected animals, smaller cells were observed, with diameters between 45 and 85 µm and more widely spaced, accompanied by a greater number of glial cells, characterized by smaller and intensely basophilic nuclei. In these tissues, abundant vasodilation, and the presence of pyknotic bodies (neurons that exhibit condensation of their cytoplasm) were observed, evidencing apoptotic processes involving the condensation of the nucleus and cytoplasm (Figure 2d).

**Figure 3:**
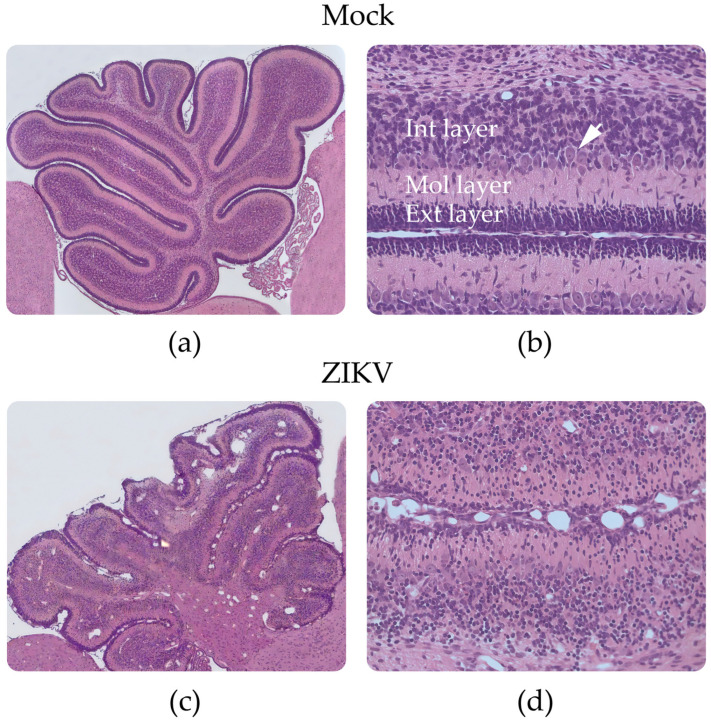
Histological findings in the cerebellum of control and Zika-infected mice: (**a**,**b**) control mouse and (**c**,**d**) infected mouse. (**b**) Detail from a control mouse; Purkinje cell layer (arrow). (**d**) Detail from an infected mouse, note alterations in the structure of the cerebellar layers. Images (**a**,**c**) (10X), (**b**,**d**) 40X.

Analyses of the cerebellum from 10 dpi mock mice showed development of the 10 cerebellar folia and the definition of four cortical layers: external granular layer, molecular layer, Purkinje cell layer, and inner granular layer (Figure 3a,b). In the group of infected mice, these same cerebellar folia were observed, but with a disorganized pattern in the four mentioned layers and with greater tissue damage toward folia VI, VII, and VIII (Figure 3c,d), where there was a cerebellar hypoplasia pattern. The external granular, Purkinje cell, and internal granular layers from the infected mice had a lower number of cells, contrary to what was observed in the molecular layer, where a greater number of cells were observed compared with that in the controls. In addition, an altered distribution pattern was observed in the Purkinje cell layer, and the presence of dilated blood vessels was noticeable in the internal granular layer from the infected animals (Figure 3d).

### 3.3. Distribution of the ZIKV Antigen, Genome, and Viral Load

**Figure 4:**
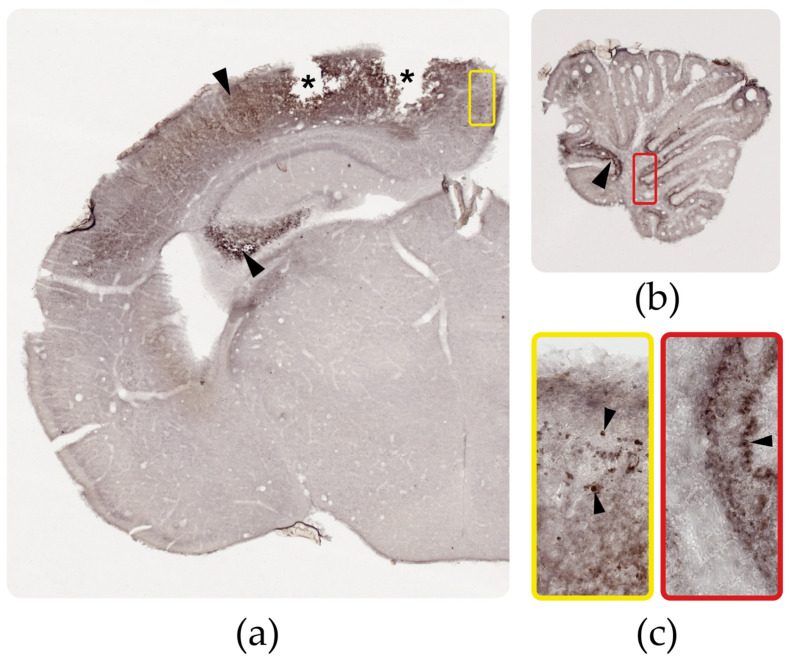
Immunoreactivity for ZIKV in the cortex and cerebellum from infected animals. (**a**) Distribution of the ZIKV antigen (arrowhead) in the cortex from infected mice (5X). The asterisks indicate the cortical areas of the brains in which calcifications were formed; in these areas the tissue was whitish and brittle and, therefore, not very resistant to cuts without a medium support, such as paraffin. (**b**) Distribution of the ZIKV antigen (arrowhead) in the cerebellum from infected mice (5X). (**c**) Greater detail from the cortex in the yellow box and cerebellum in the red box (40X). Immunostaining was performed on the vibratome cuts and revealed with DAB-Nickel.

Immunohistochemistry to detect the ZIKV antigen revealed marking in the cingulate, motor, and somatosensory cortex, in the CA3 region of the molecular layer of the hippocampus and in the molecular, granular, and internal layers, and Purkinje cells in the cerebellum (Figure 4). When comparing the dispersion of the viral antigen between the cerebral cortex and the cerebellum, the highest antigenic distribution was observed in the cerebral cortex. The results were similar to those observed during the viral copy assays performed for the two brain areas, with 22,295.2 viral copies/ng in the cerebral cortex and 6256.7 viral copies/ng in the cerebellum (Figure S1).

### 3.4. Expression of Neurodevelopmental Markers in the Cortex and Cerebellum

**Figure 5:**
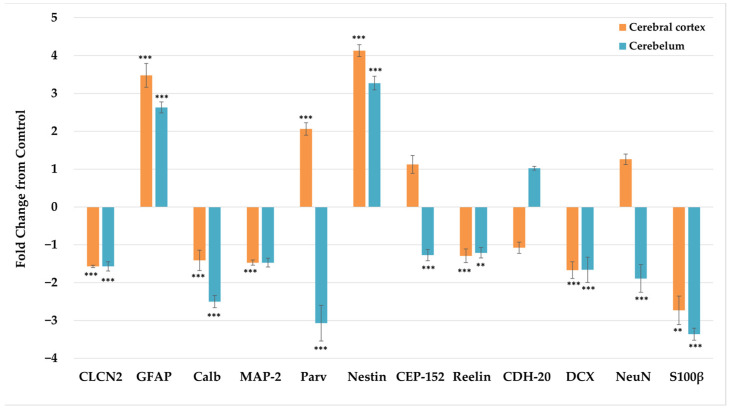
Comparison of relative gene expression in terms of the fold change as measured by qPCR. Asterisks above the qPCR values indicate significant differences between the control (mock) and infected mice. Data were analyzed with the Livak method using the efficiency calculated in each qRT-PCR run in LinRegPCR software (version 2020.0). The mock data are considered baseline, that is, zero. The data obtained for the mock and ZIKV groups for each marker were compared by means of the Wilcoxon–Mann–Whitney U test and Student’s *t* test from the results obtained to determine the normality criteria. The error bars represent the standard deviation. *p* < 0.01 (**); *p* < 0.001 (***).

The qRT-PCR assays on the cortex and cerebellum showed a strong tendency toward downregulation except for GFAP and nestin, where an increase was observed in both areas analyzed (Figure 5). The results for some genes varied between areas; for example, Parv was upregulated in the cortex and downregulated in the cerebellum. The fold change for each gene analyzed and the average efficiency per run are shown in Table S1 and Figure 5. These data were also analyzed using the GED method [77] (Table S3). In the cortex and cerebellum, the two qRT-PCR analysis methods presented the same trend for the genes: CLCN2, GFAP, Parv, nestin, reelin, DCX, NeuN, and S100β, but the trends differed for Calb and CEP-152 in the cortex and for MAP-2 and CEP-152 in the cerebellum (Table S3). However, statistical analyses were performed with the results from the Livak method because this method allows the normalization of Ct data obtained by the qRT-PCR.

**Figure 6:**
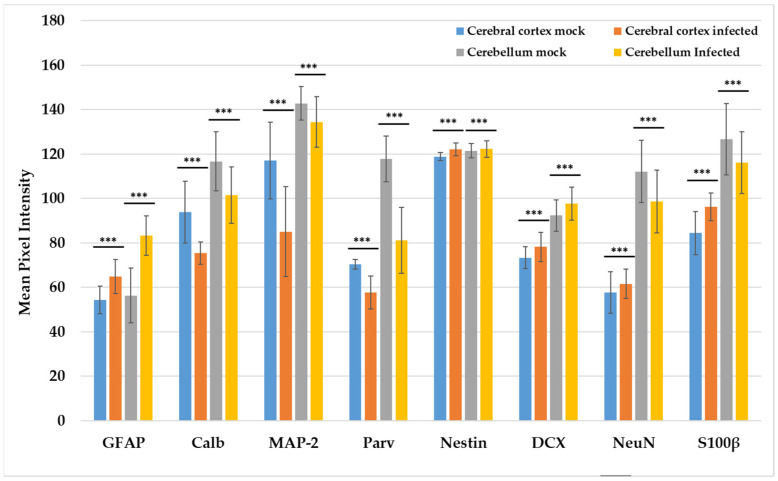
Densitometry analysis of the immunohistochemical results for different markers in the cortex and cerebellum. The analysis estimated the average grey tone from color images transformed into 8-bit images. The images of the cerebral cortex corresponded to areas spanning the different layers of the cortex in a sagittal orientation of the tissue. Similarly, the cerebellum images belonged to sagittal slices of tissue; however, the region selected for analysis along the different cerebellar folia varied according to the marker assessed, as follows: S100β, MAP-2, Parv, Nestin, Calb, and GFAP were analyzed in the molecular layer, while NeuN and DCX were analyzed in the inner granular layer. All the data were analyzed by the Wilcoxon–Mann–Whitney U test. The error bars represent the standard deviation. *p* < 0.001 (***).

**Figure 7:**
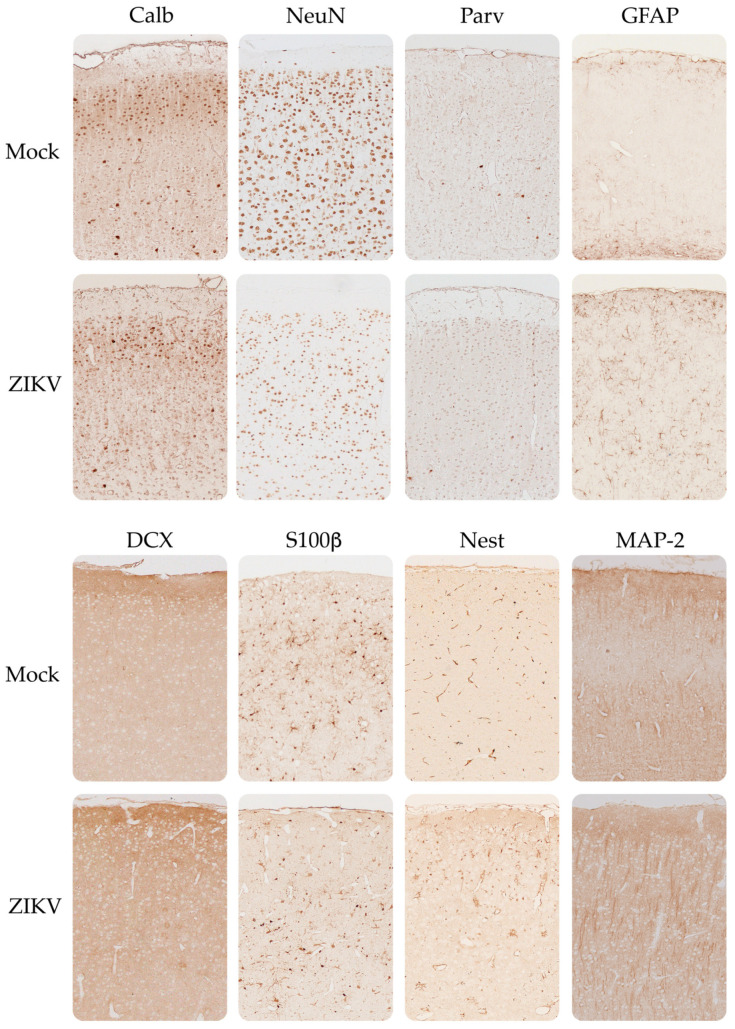
Immunohistochemical distribution of neurodevelopmental markers in the mouse cerebral cortex. Mock (cortex from control mice) and ZIKV (cortex from Zika-infected mice). 10X.

**Figure 8:**
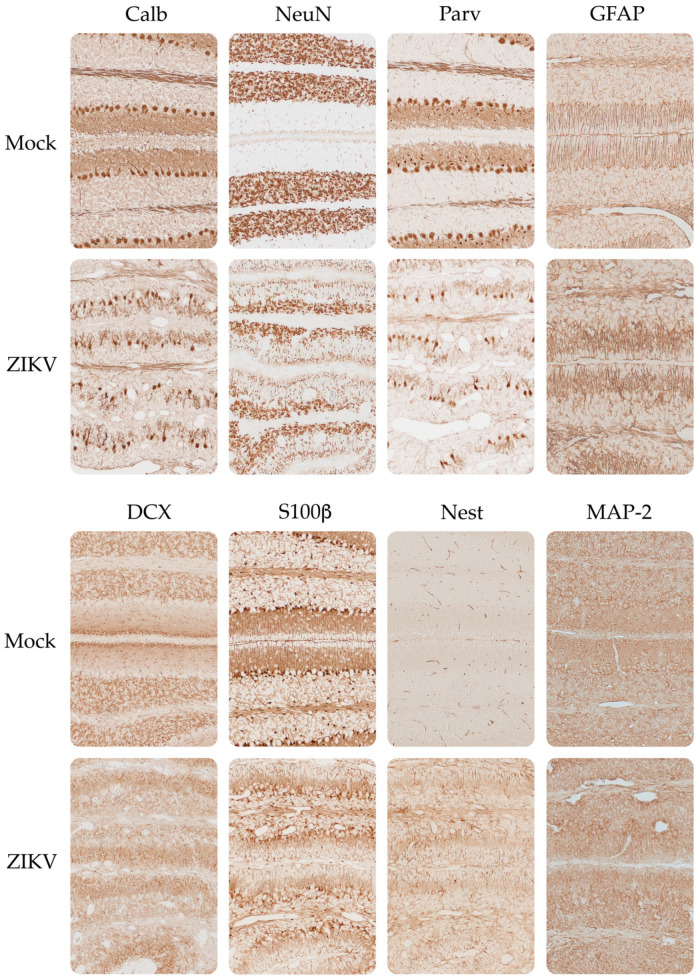
Immunohistochemical distribution of neurodevelopmental markers in the mouse cerebellum. Note the alteration in the Purkinje cell layer (Calb and Parv staining) and the loss of immunoreactivity in the inner granular layer (NeuN and DCX staining). Note the alteration of the neuronal cytoarchitecture of the external and internal granular layers (DCX staining). 10X.

The optical densitometry results for the immunohistochemical tests of the evaluated neurodevelopmental markers showed the same trend as the qRT-PCR results (Figure 6 and Table S2), except for the marker S100β, which was increased in the cerebral cortex in sections of the tissues from the mice infected with ZIKV. Regarding the neuronal cytoarchitecture, all the evaluated markers presented a uniform distribution through the layers of the cerebral cortex (Figure 7), as indicated by H&E staining. In contrast, the cerebellum from the infected mice (Figure 8) presented alterations in the distribution of the Purkinje neuron layer (IHC of Calb and Parv), in which spaces were observed between the cells and there was a smaller number of cells, as well as a strong decrease in the number of dendrites in the molecular layer. A smaller number of basket and stellate cells (Parv+) were also observed in the molecular layer, as were alterations in the cytoarchitecture and staining of the inner granular layer, appearing disorganized and scattered compared to the controls (IHC of NeuN and DCX).

## 4. Discussion

During the 2016 ZIKV epidemic, we were able to isolate and purify a Colombian strain of ZIKV from the serum of a 9-week pregnant woman with febrile symptoms and a positive PCR for this virus. This strain was able to produce neurological signs in immunocompetent neonatal BALB/c mice when inoculated by the IP route without the need to neuroadapt the ZIKV strain (Gen Bank code MH544701.2) [41,79], as required for experimental studies on the neuropathogenesis of other ZIKV strains and other flaviviruses [45,50]. In this study, we used this viral strain and observed weight loss and different neurological signs from 6 dpi, with severe paralysis or death at 11 dpi.

The neurological signs observed in ZIKV-infected mice have also been reported in other models of neonatal infection inoculated via IP or SC [34,57,80]. Wu et al. 2018, after inoculating immunocompetent infant mice with an African strain (MR-766) of ZIKV and a viral dose similar to that reported by us, showed the onset of clinical signs between the second and fifth dpi, and severe paralysis or death on the second or third day after the onset of clinical signs [80].

Fernandes et al. 2017 also reported neurological signs like ours after inoculating 1-day postnatal Swiss mice with a Brazilian (SPH-2015) ZIKV strain by IC injection, but not by the SC route. For mice injected by the IC route, the authors reported the onset of signs of disease at 6 dpi and for the SC at 12 dpi. The study did not indicate the viral load used, but reported a massive neuronal loss in the cerebral cortex and the appearance of pyknotic bodies for the model inoculated by the IC route, with minor changes for SC inoculation [50]. We also evidenced pyknotic bodies in the cerebral cortex and the massive neuronal loss only at the cerebellum level.

The differences in the days for the onset of clinical signs in the models of ZIKV infection may be due, among others, to the different routes of inoculation or to the viral strains used. The first is because the IC injection affects the cerebral cortex more severely because this is the area closest to the route commonly used for this type of inoculation [45,50], and the second is because the strains of African origin apparently generate a higher degree of lethality in relation to strains of Asian origin. This characteristic of less early cell damage or impact in strains of Asian origin could contribute to the ability of the virus to cause chronic infections in the CNS and long-term neurodevelopmental malformations [81].

We report the appearance of neurological signs that could be associated with the arrival of ZIKV in the CNS and probably with changes in the expression of the neurodevelopmental markers. After evaluating the cerebral cortex and cerebellum of infected mice we found an imbalance in the mRNA expression and immunoreactivity of different neurodevelopmental markers in the two brain areas evaluated, especially in the cerebellum, where we found a marked tendency toward downregulation.

In relation to the markers evaluated, we found increases in GFAP and nestin expression in the two brain areas evaluated by qRT-PCR and immunohistochemistry, with the increase in GFAP being possible evidence of the predominance of the differentiation of the astroglial phenotype, a previously reported finding in in vitro studies of ZIKV [82]. This becomes relevant in models of prenatal or early postnatal ZIKV infection because astrocytes have receptors that allow them to be resilient to viral infection and act as platforms for viral replication, more efficiently than neurons [27,83,84].

The correct formation of brain structures depends on the relationship between the genes that promote stem cell formation and those that promote neurogenic division [85]. Symmetric divisions are primarily responsible for stem cell development, while asymmetric divisions or differentiation are responsible for neurogenic division [85]. These divisions can be strongly regulated by the centrosomal proteins that determine the stability of the cilia, whose alteration could favor neural delamination and premature neurogenesis, a condition that has been reported in studies on ZIKV infection [86].

The CEP-152 mRNA was downregulated in the cerebellum, with no significant changes in the cerebral cortex. The protein encoded by this gene is involved in the biogenesis of centrioles and in centrosomal function, and mutations in this gene have previously been associated with Seckel syndrome, which is characterized by the presence of microcephaly and dwarfism [86]. This protein forms a complex with CEP-63 to control the formation of centrosomes and, thus, alterations in the protein decrease the pool of neural precursors necessary for the growth of the human brain, generating alterations in the size of the cerebral cortex or other malformations reported in the microcephaly phenotype [87].

The involvement of centrosomal proteins has also been evidenced in other models of ZIKV infection. Saade et al. [22] found a decrease in the expression of the centriole protein CEP-164 in biopsies of human fetuses positive for ZIKV and in neural progenitor cells (NPCs) transfected with the viral replication protein NS5 [22]. These biopsies showed a shortening of the cilia in the ventricular cavity, similar to biopsies obtained from chicken embryos transfected with the same viral protein. The authors postulate that the shortening of the cilia could be a sign of neural delamination, a hypothesis that is supported by an increase in the levels of the TIS-21 gene, a marker associated with decreased stem cell formation and increased neurogenic division [88]. These results are evidence that an imbalance in centrosome proteins could promote premature differentiation.

The hypothesis of premature differentiation postulated during ZIKV infection, associated with an imbalance in centrosomal proteins, has also been supported by Gabriel et al. [89], who, after infecting NPC cells, found alterations in the recruitment of the centrosomal proteins CEP-152, pericentrin (PCNT), and centrosomal P4.1-associated protein (CPAP), as well as a reduction in the levels of CEP-164. In that study, the authors also observed changes in the orientation of the brain organoids infected by ZIKV, with a tendency to the vertical plane in the infected organoids, while a tendency to the horizontal plane occurred in the mock, thus indicating a situation that favors asymmetric division, i.e., those where differentiation is promoted.

As previously mentioned, there were increased levels of nestin mRNA and protein in the cerebral cortex and cerebellum, in the tissues of ZIKV-infected mice. This protein is part of the intermediate class VI filaments present in neural stem cells, neurogenic cells, and precursors of radial glia, among other cells [90]. Nestin is also known as a marker of progenitor cells required for the survival and self-renewal of CNS cells; nestin is downregulated when progenitor cells differentiate into neurons or glial cells, leading to an increase in the expression of GFAP, a marker of astrocytes. In the adult brain, nestin remains in the areas with progenitor cells of the neurogenic zones in the forebrain [91].

The role of nestin as an ostensible marker of neural progenitor cells under physiological conditions has been established [90,91]. However, Krishnasamy et al. [92] found that the expression of this marker can change under conditions of neuroinflammation. The authors reported that nestin can be expressed in mature astrocytes or in brain microglia during ischemia or after treatment with lipopolysaccharide (LPS), thereby favoring differentiation toward gliosis. This last finding is similar to our results because we found an increase in GFAP expression, which could be interpreted as a cooperative mechanism used by the virus to promote astroglial differentiation to create an environment that favors its replication and viral dissemination [83].

The expression of some markers was different using different techniques; for example, DCX was shown to decrease when measured by qRT-PCR and to increase when measured by immunohistochemistry (Figure 5 and Figure 6). The increase in DCX expression could be a viral dissemination strategy, as it has an important role in the organization and stabilization of microtubules [93], which could become relevant when considering that these organelles serve as platforms for the mobilization of the viral vesicles of ZIKV and for most viruses that have a nucleocapsid [34]. DCX expression may also increase due to neuroinflammation events; for example, in models of cerebral ischemia in mice, colocalization between the DCX and Toll-like receptor 2 (TLR2) and between this receptor and cells positive for the microglial marker IBA-1, have been described [94]. This protein participates in neuronal migration processes during neurodevelopment; therefore, alterations or changes in expression could be associated with misarrangement of the cerebellar layers found in the tissues of mice infected with ZIKV [95].

Other models of ZIKV infection have found a decrease in DCX at the mRNA and protein levels. Jiang et al. found a decrease in DCX levels after infecting NPC cells and fetal mouse brains and speculated that the loss of expression could be attributed to the presence of viral proteins of ZIKV, i.e., NS4A and NS5 [23]. Loss of DCX has been reported in prenatal models and in human progenitor cells infected with the flavivirus; however, the effects of ZIKV on the expression of DCX could vary based on the stage evaluated, as we report in the present study and as commonly described in the different ZIKV infection models for other markers evaluated during viral infection [22].

Unlike DCX, MAP-2, which is a microtubule-associated protein, was shown to decrease in the two brain areas using the two techniques evaluated. The loss or increase in MAP-2 levels could be associated with damage to postmitotic dendrites because their function is associated with maintaining their stability [96]. The association between dendritic pathology and changes in the expression of MAP-2 has been postulated in brain and spinal cord studies on rabies virus-infected mice [70,97,98] and in other pathologies, such as cerebral ischemia, Rett syndrome, Down syndrome, and epilepsy [96,99,100]. Therefore, alterations in the levels of MAP-2 expression could contribute to damage to the connectivity of projection cells, as occurs with the pyramidal neurons present in the cerebral cortex and Purkinje cells in the cerebellum.

Calb and Parv were other markers with alterations in expression levels. Both markers showed a tendency to be downregulated; however, for Parv, an increase in the cerebral cortex was observed when measured by both techniques. These proteins are commonly known as calcium binding proteins (CaBP), and their function is to act as calcium regulators, transporting calcium to places with high metabolic demand in the cell [101], and they have the ability to help model the synaptic response and neuronal excitability; therefore, an imbalance or dysfunction has been associated with neuronal degeneration processes [101,102].

After ZIKV infection, a loss of Calb immunoreactivity has been reported in hippocampal organotypic cultures in dentate gyrus granule cells [103] and in the Purkinje neurons of the cerebellum in neonatal mice inoculated subcutaneously. We found loss of Calb and Parv immunoreactivity in the Purkinje cell layer and in the cerebellum molecular layer in the group of mice infected with ZIKV. The loss of these two proteins could be associated with dysfunction of the GABAergic system during ZIKV infection; Purkinje neurons and interneurons present in the molecular layer (basket and stellate cells) that express these two CaBP release the neurotransmitter GABA, the main amino acid that regulates inhibition in the brain and is also actively involved during neurodevelopment and neuronal differentiation [104].

We found increased immunoreactivity to Parv in the cerebral cortex from mice infected with ZIKV. This increase has also been reported in studies on mice intramuscularly inoculated with rabies virus [105]. Parv expression levels may increase as a neuroprotective mechanism [106], and this could, among other things, contribute to reducing the damage or morphological alterations observed in the cerebral cortex.

Changes in the expression of Parv, Calb, and CLCN2 in the cerebellum could be associated with clinical signs, such as tremor and uncoordinated gait, observed in mice infected with ZIKV. These movement disorders may be associated with changes due to cerebellar ataxia linked to dysfunction or loss of these three markers; similar manifestations have been observed in other studies on Zika [107]. In addition, in mouse models of spinocerebellar ataxia, the loss of Calb and Parv has been reported in Purkinje neurons [108]. Mutations in CLCN2 have also been associated with cerebellar ataxia occurring during dystrophic leukoencephalopathy, a neurodevelopmental disorder characterized by cerebellar ataxia, spasticity, optic neuropathy, and chorioretinopathy with visual defects [109,110].

CLC-2 is a voltage-gated chloride channel encoded by the CLCN2 gene (Clcn2 mouse). This channel maintains the balance of chloride ions by allowing the outflow of Cl- from neurons and preventing excessive accumulation within the cell during inhibitory transmission [111]. Knockout mice for this gene exhibit blindness, retinal damage, and myelin vacuolization in brain tissue and the spinal cord [112,113]. Dysfunction of this protein has also been associated with neurodevelopmental disorders, Tourette syndrome, and idiopathic epilepsy; however, a direct relationship between the latter and alterations in the channel has not yet been described [109].

The expression of CLC-2 regulates changes in excitatory signals mediated by the activation of the GABA-A receptor because the Cl- gradient is passively modified, a phenomenon that occurs between the late embryonic and early neonatal stages [114]. A change in CLC-2 expression can alter synaptogenesis and neurogenesis processes mediated by GABAergic signaling during these stages. This phenomenon has been observed in a model of early exposure to ethanol in rats, in which a decrease in ClC-2 expression was observed in both the cerebral cortex and the cerebellum, associated with an alteration in the inhibitory signals of GABA during synaptogenesis [115]. In this sense, ZIKV infection can alter GABA neurotransmission and associated neurodevelopmental processes.

The decrease in the expression of NeuN could be correlated with the strong impairment observed in the integrity of the tissue observed with H&E staining and immunohistochemistry. These alterations suggest that infection may generate cell death in the cerebellum, given the loss of Calb, Parv, and NeuN immunoreactivity. Some studies on ZIKV infection models have reported the death of cells in the inner granular layer and of Purkinje cells in the cerebellum. In studies on two different strains of the ZIKV virus (French and Brazilian Polynesia) in neonatal mice inoculated by IP injection, induction of apoptosis was reported in the cerebellum, with severe alterations in the morphology of the cerebellar folia and cerebellar hypoplasia [34].

In a neonatal C57BL/6 mouse model inoculated with the PRVABC59 strain of ZIKV by SC injection, neuron infection in the cerebellum was associated with apoptosis and the infiltration of immune cells, as well as calcification and decreased cerebellar volume [57]. In this study, in addition, loss of Purkinje cells and their neurites inferred by loss of GAD67+ and neurofilament-H protein labeling was observed [57], this finding was also observed in our model after immunostaining with Calb and Parv. Cell death resulting from ZIKV infection has also been observed in neural stem cells used as a model of neurodevelopment [116], as well as in neural progenitor cells in organoids that served as models for the first trimester of cortex development [117]; both studies generated approaches to elucidate the mechanisms of ZIKV microcephaly observed in humans.

Morphological and molecular changes found in ZIKV-infected mice may vary depending on the age of infection. The presence of intracerebral calcifications is commonly reported during human ZIKV microcephaly cases, but these apparently do not occur in prenatal inoculation mouse models unless the infection is allowed to progress to later postnatal stages [11,23,39]. For example, C57BL/6 Ifnar1−/− mice inoculated at E6.5 or E7.5 and sacrificed between E13.5 and E16.5 did not exhibit them [11], while offspring of C57BL/6 wild-type mice inoculated at E15 intraamniotically showed calcified brains when infection was allowed to progress until postnatal day 40 [47]. These findings could indicate that calcifications mainly originate from neuronal damage and this damage is not evident if observations are made near the onset of neurogenesis, a stage that for the mouse would correspond to E10 [118].

On the other hand, the few molecular changes observed in the cerebral cortex found in our study can be attributed, among others, to the limited role of neural progenitor cells during the infection stage evaluated, since neurogenesis is almost complete [34] in postnatal models. The cerebral cortex cells have already migrated, mostly before birth, in rodents [119]. In this sense, the postnatal infection does not manifest profound alterations in the structure of the cerebral cortex [119,120], as observed in our model.

The growth in the cerebellar cortex occurs postnatally in both humans and mice due to the neuronal progenitor cells (GNPs) present in the external granular layer (EGL), whose proliferation is stimulated by sonic hedgehog genes (Shh) present in the Purkinje neurons. The EGL disappears on postnatal day 20 in mice [121,122], whereas, in humans, a variable range of loss for this layer has been reported between the 11th and 20th postnatal months [123,124,125]. GNPs in turn secrete reelin which regulates the correct localization of Purkinje neurons [126]. The EGL is a site of cell proliferation in the cerebellum in different animals and humans. This layer is an area of progenitor cells, which makes it sensitive to X-ray radiation and viral infections [121,122]. The cells in this layer will give rise to the glutamatergic granule cells present in the inner granular layer, whose axons bifurcate into parallel fibers to contact the Purkinje branches projected in the molecular layer [127,128]. EGL disorders produce alterations in the distribution of interneurons in the molecular layer, heterotypic distribution of granule cells in the internal granular layer, and disorganization of Purkinje cells with misorientation in the bifurcation of their dendrites [122].

In this study, the qRT-PCR indicated a decrease in reelin in the cerebellum and alterations in the external granular layer (Figure 3 and Figure 5); therefore, this could be another marker whose decrease contributes to cerebellar hypoplasia and the poor localization of the cerebellar layers in the tissues of animals and humans infected with ZIKV [6,47,129]. As part of the cerebellar development occurs after birth, this encephalic zone is more vulnerable to environmental disturbances [120,122,130], which is strongly evidenced in the immunohistochemical labeling of reelin and the other markers in the cerebellum of mice infected with ZIKV.

Reelin is also expressed in the horizontal cells of Cajal in layer I, where it regulates the correct orientation of the pyramidal neurons [131,132]. Therefore, dysfunction or modifications in the expression levels of reelin could be associated with alterations in pyramidal dendrites or dendrite pathologies that could appear during ZIKV infection; however, studies are necessary to corroborate the dendritic pathology associated with the dysfunction of this protein or of microtubule proteins, such as MAP-2.

The mRNA and immunostaining levels of S100β also decreased at the cerebellar level. S100β is a calcium-binding protein and is found in astrocytes, oligodendrocytes, and Schwann cells [133,134]. Among its main functions, it promotes the extension of neurites, protects against oxidative damage caused by copper ions and the neurotrophic and mitogenic activity that promotes the proliferation of glial cells, and the increase in Ca^2+^ in these cells and in neurons [133].

The inhibition or downregulation of S100β could affect glial cell proliferation [133] and be indicative of the increase in glutamate levels because glutamate reuptake inhibitors increase the expression of S100β, and extracellular concentrations of glutamate above physiological levels inhibit its release [135]. As part of other studies carried out by our working group, we found an increase in glutamate immunoreactivity in the cerebellum (the results are not presented), a finding that could be associated with the loss of S100β. Tramontina et al. postulated that the inhibition of S100β, due to high levels of glutamate in astrocytes, could be mediated by glutamate aspartate transporters (GLAST) and type II metabotropic glutamate receptors through an inhibitory mechanism of cAMP levels [136]. As we found an increase in GFAP levels, the decrease in the levels of S100β are likely not due to a decrease in the astroglial population; therefore, we associate the loss to an imbalance in glutamate metabolism in this brain area in ZIKV-infected mice.

**Figure 9:**
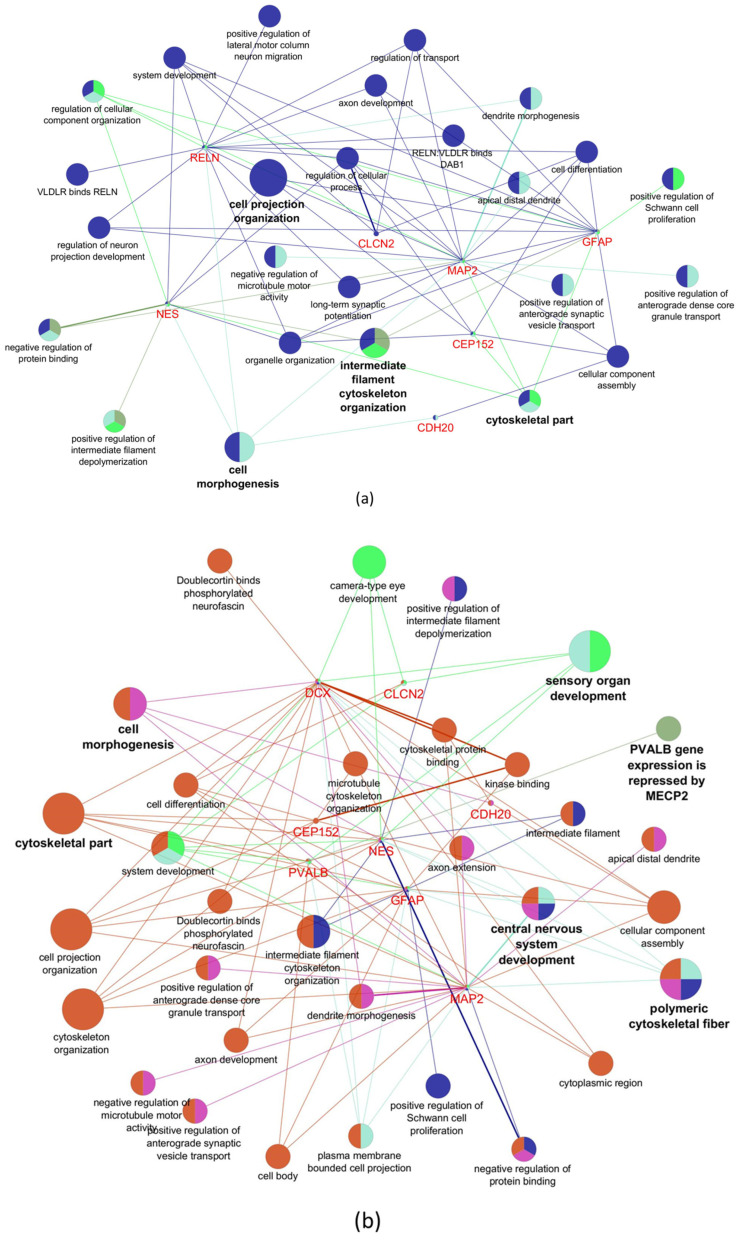

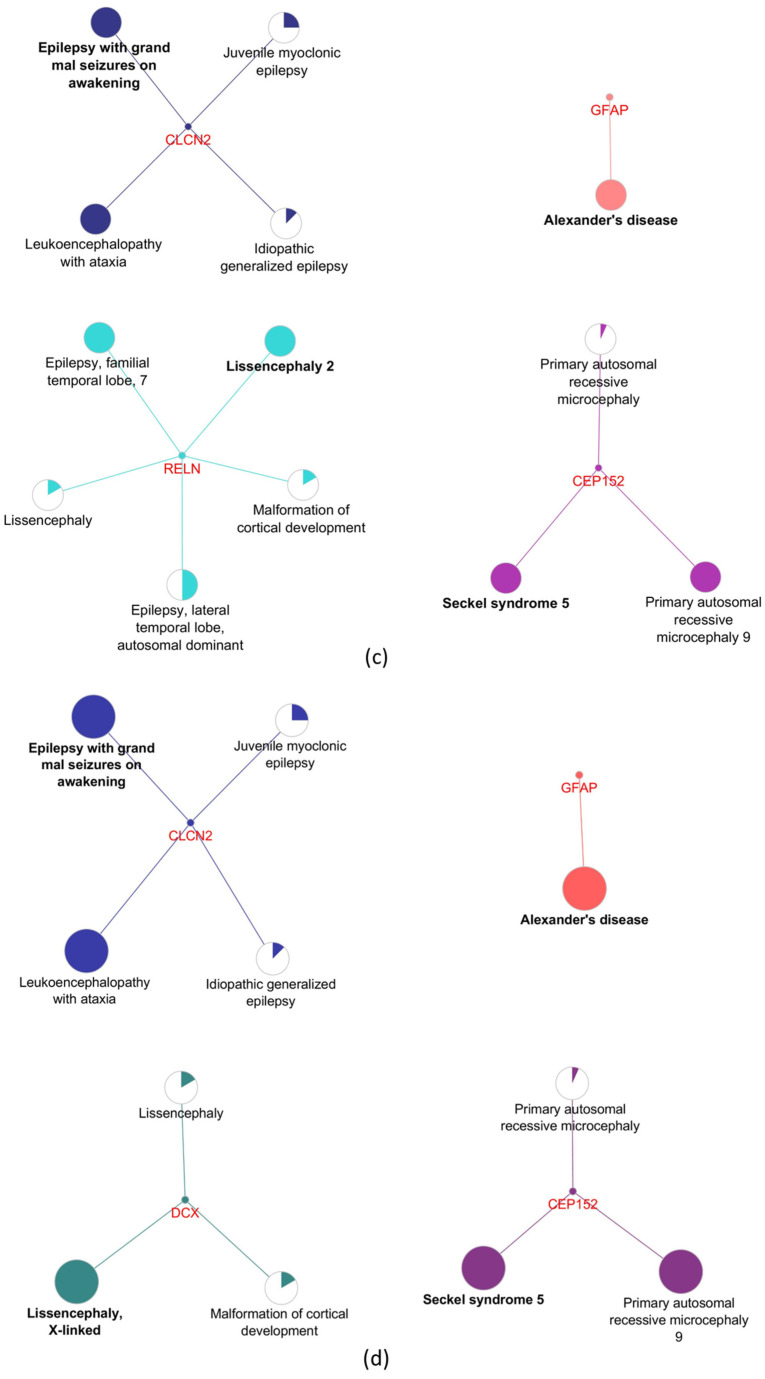
Network analysis of dysregulated genes in the cortex and cerebellum. Biological processes potentially altered by the dysregulated genes in the cortex (**a**) and cerebellum (**b**). Human diseases potentially associated with dysregulated genes in the cortex (**c**) and cerebellum (**d**). Colors and bold text indicate the main gene-enriched terms. In panel (**a**), blue for cell projection organization; olive for intermediate filament cytoskeleton; green for cytoskeletal; and aquamarine for cell morphogenesis. In panel (**b**), magenta for cytoskeletal; olive for Parv gene expression repressed by MECP2; green for sensory organ development; aquamarine for polymeric cytoskeletal fiber; blue for central nervous system development; and magenta for cell morphogenesis. Panels (**c**,**d**) represent the percentage of association for each gene with human diseases.

Finally, after reviewing the functions of each marker evaluated and after performing a network analysis on the dysregulated genes in the cortex and cerebellum of ZIKV-infected mice in our study, we found that the biological processes involved in the morphogenesis of these cerebral structures are potentially altered during the infection by the flavivirus, such as, dendrite morphogenesis, axon development, positive regulation of Schwann cell proliferation, cell morphogenesis, cell projection organization, and intermediate filament cytoskeleton organization (Figure 9a,b). However, some biological processes appear to be specifically affected in each brain structure. The biological processes with the greatest gene convergence of the markers evaluated in the cerebral cortex were associated with the development of neuronal projection and lateral migration of the motor cortex (Figure 9a), whereas in the cerebellum there was greater convergence of genes linked to the development of the eye chamber and sensory organs (Figure 9b). Altered genes in the cerebral cortex and cerebellum are associated with demyelinating phenotypes, various types of epilepsy, Alexander disease, and other conditions related to microcephaly, such as lissencephaly and Seckel syndrome (Figure 9c,d).

## 5. Conclusions

In this study, we found changes in the mRNA levels and immunoreactivity of markers associated with neurodevelopment in immunocompetent neonatal mice infected with ZIKV. Several of the evaluated markers presented alterations in the cerebral cortex and the cerebellum, the latter being the main area affected in the histological and molecular analyses. The altered markers indicate that during ZIKV infection dendritic pathology may occur in the projection neurons of the cortex and cerebellum, with an imbalance in intracellular calcium and chloride flux, changes in proteins associated with neuronal proliferation and differentiation whose alterations have been linked to neurodevelopmental dysfunction and malformations, and probably to dysfunction in the metabolism of the neurotransmitters GABA and glutamate. This hypothesis and dendritic pathology need to be tested. It is possible that the evaluated markers serve as viral binding or replication targets, which is why it is necessary to develop protein interaction studies on these markers and the proteins involved in the replication of ZIKV to find new targets that could be used for therapeutic purposes to reduce or eliminate malformations that occur during infection by this flavivirus.

## Figures and Tables

**Table 1:** Antibodies used in the immunohistochemical assays.

Antibody	Supplier	Catalog Number	Host	Clonality	Dilution
Anti-NeuN	Cell Signaling Technology	24307S	Rabbit	Monoclonal	1:600
Anti-Calbindin	Sigma-Aldrich	C9848	Mouse	Monoclonal	1:400
Anti-Parvalbumin	Sigma-Aldrich	P3088	Mouse	Monoclonal	1:2000
Anti-S100	Abcam	ab868	Rabbit	Polyclonal	1:400
Anti-Nestin	Abcam	ab6142	Mouse	Monoclonal	1:100
Anti-DCX	Abcam	ab18723	Rabbit	Polyclonal	1:10,000
Anti-MAP-2	Santa Cruz	Sc 20172	Rabbit	Polyclonal	1:2500
Anti-GFAP	Dako	Z0334	Rabbit	Polyclonal	1:1000
Anti-ZIKV	CDC’s Division of Vector-Borne Diseases	NA	Mouse	Polyclonal	1:1000
	Cell Morphology-INS	NA	Rabbit	Polyclonal	

**Table 2:** Primers and probes for the differential expression assays.

Gene Symbol or Genome Region	Primer Sequence (5′-3′)	TM (°C)	Size Amplicon (bp)	GenBank Accession No.	Author
* GAPDH	F: AGGTCGGTGTGAACGGATTTG	62.6	95	(NM_017008)	
	R: GGGGTCGTTGATGGCAACA	62.6			
** CDH20	F: GGGACCACAACAGTCAACATC	55	191	(AF007116)	[72]
	R: GCACCATCTCCATCCACAAT	55			
** DCX	F: CATTTTGACGAACGAGACAAAGC	60.8	63	(NM_010025)	
	R: TGGAAGTCCATTCATCCGTGA	60.9			
** RELN	F: TCTCTTCGTGGGTTGTTTCC	58.4	136	(NM_011261)	
	R: GCATGGTTCAGCCTAGAGTG	60.5			
** GFAP	F: CGGAGACGCATCACCTCTG	62.1	120	(NM_001131020)	
	R: TGGAGGAGTCATTCGAGACAA	60.2			
** NES	F: CCCTGAAGTCGAGGAGCTG	61.4	166	(NM_016701)	[73]
	R: CTGCTGCACCTCTAAGCGA	61.7			
** NeuN	F: GGCAAATGTTCGGGCAATTCG	63	160	(NM_001039167)	[74]
	R: TCAATTTTCCGTCCCTCTACGAT	61.1			
** CALB1	F: GGCTTCATTTCGACGCTGAC	61.7	184	(NM_009788)	
	R: ACGTGAGCCAACTCTACAATTC	60.3			
** MAP2	F: CCACTGCCGGACCTGAAGAATG	60.3	164	(NM001039934)	
	R: CCCCCAGCAGAATGTTTGATGTTA	59			
** CEP152	F: CCCTTTGCAGAACGCCACCAC	61.2	197	(NM001081091)	
	R: CCGCAACATTCCGCTTTTACCA	61.9			
** CLCN2	F: ATGTATGGCCGGTACACTCAG	61.3	163	(NM_009900)	
	R: AACAAATGCGACATCTGGCAC	59.4			
** S100B	F: GGTGACAAGCACAAGCTGAA	58.4	139	(NM_009115)	
	R: ACTCCCCATCCCCATCTTCG	62.5			

* Normalizer gene; ** neural markers evaluated.

## Data Availability

The analysis data were uploaded to a repository and the link was added. https://data.mendeley.com/datasets/p4ykvx9rjd/1 (accessed on 30 May 2023).

## Supplementary Materials

The following supporting information can be downloaded at https://www.mdpi.com/article/10.3390/v15081632/s1: Figure S1. Change in body weight and viral load in cerebral cortex and cerebellum in mock and ZIKV-inoculated mice; Table S1. Fold change (vs. mock) and percentage of efficiency of the neurodevelopmental marker genes evaluated by qRT-PCR; Table S2. Optical density levels for neurodevelopmental markers evaluated by immunohistochemistry; Table S3. Relative expression of developmental marker genes for cerebral cortex and cerebellum in ZIKV-infected mice.
