# Supplementary material for: Morphological and Molecular Changes in the Cortex and Cerebellum of Immunocompetent Mice Infected with Zika Virus
Source: Viruses. 2023 Jul 27;15(8):1632. doi: 10.3390/v15081632 (PMC10458311; doi:10.3390/v15081632)
Supplement: Supplementary file 1 [file viruses-15-01632-s001.zip › viruses-2455318-supplementary/Figure S1 corrected (19-07-2023).pdf]

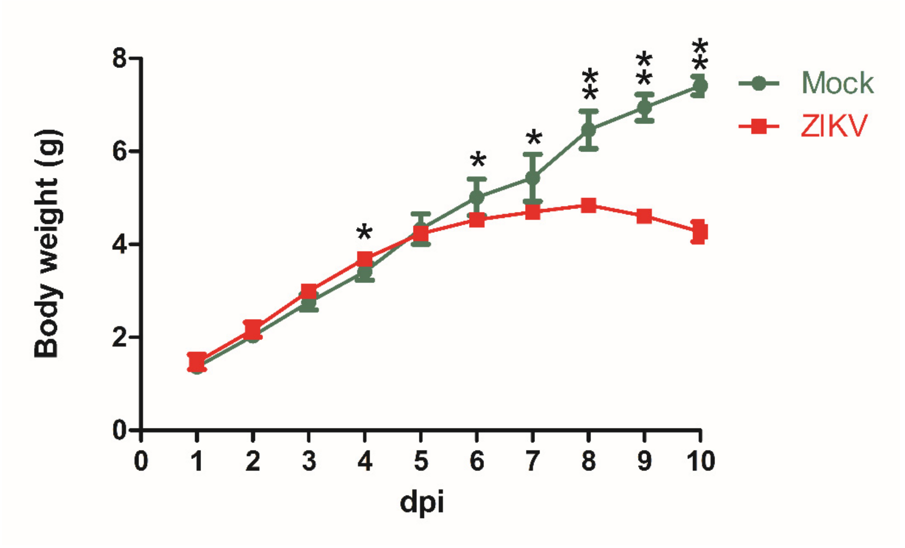

(a)

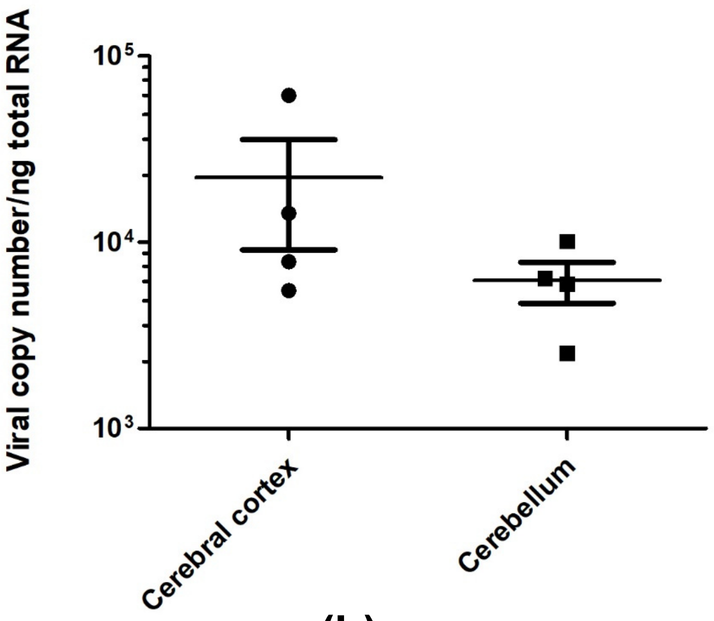

(b)

Figura S1. Change in body weight and viral load in the cerebral cortex and cerebellum in mock and ZIKV-inoculated mice. (a) Mean body weights of 1-day-old postnatal BALB/c mice inoculated with ZIKV (n=7) and virus-free solution (mock) (n=7), up to the time of euthanasia. Weights were analyzed by Wilcoxon-Mann-Whitney U test, except for dpi 4 and 10 which were analyzed by t-student test according to normality criteria (* $p<0.01$; ** $p<0.001$). (b) Viral load obtained by qRT-PCR in the

cerebral cortex and cerebellum of mice inoculated with ZIKV (n=4). The error bars represent the standard deviation.
