# Supplementary material for: Morphological and Molecular Changes in the Cortex and Cerebellum of Immunocompetent Mice Infected with Zika Virus
Source: Viruses. 2023 Jul 27;15(8):1632. doi: 10.3390/v15081632 (PMC10458311; doi:10.3390/v15081632)
Supplement: Supplementary file 1 [file viruses-15-01632-s001.zip › viruses-2455318-supplementary/Table S1 corrrected (19-07-2023).pdf]

Table S1. Fold change (vs mock) and percentage of efficiency of the neurodevelopmental markers genes evaluated by qRT-PCR

Brain area evaluated								
	Cerebral Cortex				Cerebellum			
**Gene name**	**Efficiency percentage**	**Fold Change**	**SD**	**P-value**	**Efficiency percentage**	**Fold Change Change**	**SD**	**P-value**
Chloride voltage-gated channel (CLCN2)	90	**-1,57**	0,03	0,0006*	87,6	**-1,57**	0,12	1,52E-8**
Glial fibrillary acidic protein (GFAP)	93	**3,48**	0,31	0,0009*	94,7	**2,63**	0,15	0,00004*
Calbindin (Calb1)	90,4	**-1,41**	0,27	0,0001**	89,3	**-2,50**	0,16	0,00004*
Microtubule associated protein 2 (MAP-2)	90,5	**-1,47**	0,07	0,0001*	106,4	**-1,47**	0,12	0,776915**
Parvalbumin (Parv)	88	**2,06**	0,17	1,57772E-7**	88,1	**-3,07**	0,47	<<0,0001**
Nestin (Nestin)	93,4	**4,13**	0,16	0,0004*	93,3	**3,27**	0,18	0,0009*
Centrosomal protein 152 (CEP-152)	95	**1,13**	0,24	0,189297*	94,6	**-1,27**	0,15	0,0003**
Reelin (Reelin)	88,3	**-1,29**	0,18	0,0001**	87	**-1,21**	0,14	0,0043*
Cadherin 20 (CDH-20)	98,1	**-1,08**	0,15	0,1059**	86,8	**1,03**	0,05	0,791**
Doublecortin (DCX)	94	**-1,67**	0,22	3,49259E-7**	94,5	**-1,66**	0,34	0,00002**
NeuN (NeuN)	85,5	**1,26**	0,14	0,0930*	90	**-1,89**	0,37	0,00001**
S100 calcium binding protein B (S100B)	87,6	**-2,73**	0,38	0,0027157*	83,6	**-3,36**	0,16	0,0002*

The data obtained for mock and ZIKV groups for each marker were compared using the Wilcoxon-Mann-Whitney U test (*) and the Student's t-test (**) from the results obtained to determine the normality criteria. The data correspond to the analysis of four biological samples and three technical replicates.
