# Supplementary material for: Morphological and Molecular Changes in the Cortex and Cerebellum of Immunocompetent Mice Infected with Zika Virus
Source: Viruses. 2023 Jul 27;15(8):1632. doi: 10.3390/v15081632 (PMC10458311; doi:10.3390/v15081632)
Supplement: Supplementary file 1 [file viruses-15-01632-s001.zip › viruses-2455318-supplementary/Table S2 (07-07-2023).pdf]

Table S2. Optical density levels for neurodevelopmental markers evaluated by immunoshistochemistry.

Gene name	Cerebral Cortex			Cerebellum		
	**Mean Pixel Intensity Mock ± SD**	**Mean Pixel Intensity ZIKV ± SD**	**P-value**	**Mean Pixel Intensity Mock ± SD**	**Mean Pixel Intensity ZIKV± SD**	**P-value**
Calbindin (Calb1)	93,95±13,87	75,39±5,07	<<0,001	116,73±13,23	101,56±12,72	<<0,001
Doublecortin (Dcx)	73,34±4,88	78,21±6,64	7,17 E-10	92,37±7,08	97,73±7,40	<<0,001
Glial fibrillary acidic protein (Gfap)	54,3±6,24	64,9±7,77	<<0,001	56,33±12,36	83,27±8,87	<<0,001
Microtubule associated protein 2 (Map2)	117,2±17,25	85,05±20,25	<<0,001	142,78±7,57	134,38±11,40	<<0,001
Nestin (Nes)	118,8±1,79	122,11±2,89	<<0,001	121,53±3,22	122,27±3,67	1,33E-07
NeuN (NeuN)	57,72±9,24	61,58±6,63	2,54 E-10	112,18±14,08	98,65±14,05	<<0,001
Parvalbumin (Pvalb)	70,4±2,08	57,71±7,50	<<0,001	117,84±10,29	81,18±14,85	<<0,001
S100 calcium binding protein B (S100)	84,46±9,69	96,35±6,24	<<0,001	126,67±16,14	116,14±13,82	<<0,001

Higher values in pixel intensity indicate higher immunostaining of the marker evaluated. All data were analyzed by the Wilcoxon Mann Whitney U test.
