# Supplementary material for: Morphological and Molecular Changes in the Cortex and Cerebellum of Immunocompetent Mice Infected with Zika Virus
Source: Viruses. 2023 Jul 27;15(8):1632. doi: 10.3390/v15081632 (PMC10458311; doi:10.3390/v15081632)
Supplement: Supplementary file 1 [file viruses-15-01632-s001.zip › viruses-2455318-supplementary/Table S3 (07-07-2023).pdf]

Table S3. Relative expression of developmental marker genes for cerebral cortex and cerebellum in ZIKV-infected mice.

Gene	Cerebral Cortex					Cerebellum				
	**GED (mean E)**		**GED (Individual E)**			**GED (mean E)**		**GED (Individual E)**		
	**rER**	**Fold Change**	**rER**	**Standard deviation**	**Fold Change**	**rER**	**Fold Change**	**rER**	**Standard deviation**	**Fold Change**
Chloride voltage-gated channel 2 (Clcn2)	0,69	**-1,44**	0,75	0,2	**-1,33**	0,57	**-1,75**	0,78	0,5	**-1,29**
Glial fibrillary acidic protein (Gfap)	4,21	**4,21**	5,68	1,96	**5,68**	3,04	**3,04**	4,81	4,34	**4,81**
Calbindin (Calb1)	1,06	**1,06**	1,1	0,48	**1,1**	0,38	**-2,66**	0,43	0,27	**-2,3**
Microtubule associated protein 2 (Map2)	0,63	**-1,59**	0,66	0,28	**-1,53**	1,23	**1,23**	1,47	1,22	**1,22**
Parvalbumin (Pvalb)	1,58	**1,58**	2,09	0,96	**2,09**	0,28	**-3,6**	0,33	0,1	**-3,07**
Nestin (Nes)	2,35	**2,35**	2,16	0,82	**2,16**	3,34	**3,34**	3,7	1,32	**3,7**
Centrosomal protein 152 (Cep152)	0,74	**-1,35**	0,56	0,02	**-1,77**	1,49	**1,49**	1,23	0,93	**1,23**
Reelin (Reln)	0,89	**-1,12**	0,93	0,04	**-1,08**	0,52	**-1,91**	0,91	0,63	**-1,1**
Cadherin 20 (Cdh20)	0,78	**-1,29**	0,92	0,31	**-1,09**	0,37	**-2,73**	0,59	0,55	**-1,7**
Doublecortin (Dcx)	0,59	**-1,71**	0,75	0,26	**-1,33**	0,21	**-4,85**	0,51	0,45	**-1,97**
NeuN (NeuN)	1,54	**1,54**	1,89	1,84	**1,89**	0,39	**-2,55**	0,42	0,24	**-2,37**
S100 calcium binding protein B (S100)	0,41	**-2,43**	0,38	0,04	**-2,64**	0,29	**-3,39**	0,37	0,26	**-2,72**

Relative expression ratio (rER) was calculated according to the Gene Expression Ct's Difference (GED) [79] with averaged efficiency (mean E) for each gene and individual efficiency for all samples (individual E). The average efficiency and individual efficiency were determined using LinRegPCR [77].
